# Beneath the surface: community assembly and functions of the coral skeleton microbiome

**DOI:** 10.1186/s40168-019-0762-y

**Published:** 2019-12-12

**Authors:** Francesco Ricci, Vanessa Rossetto Marcelino, Linda L. Blackall, Michael Kühl, Mónica Medina, Heroen Verbruggen

**Affiliations:** 1 School of BioSciences, University of Melbourne, Parkville, 3010 Australia; 2 Marie Bashir Institute for Infectious Diseases and Biosecurity, Sydney Medical School, Westmead Clinical School, The University of Sydney, Sydney, NSW 2006 Australia; 3 Marine Biological Section, University of Copenhagen, Strandpromenaden 5, DK-3000 Helsingør, Denmark; 4 Climate Change Cluster, University of Technology Sydney, Ultimo, NSW 2007 Australia; 5 Pennsylvania State University, University Park, PA 16802 USA

## Abstract

Coral microbial ecology is a burgeoning field, driven by the urgency of understanding coral health and slowing reef loss due to climate change. Coral resilience depends on its microbiota, and both the tissue and the underlying skeleton are home to a rich biodiversity of eukaryotic, bacterial and archaeal species that form an integral part of the coral holobiont. New techniques now enable detailed studies of the endolithic habitat, and our knowledge of the skeletal microbial community and its eco-physiology is increasing rapidly, with multiple lines of evidence for the importance of the skeletal microbiota in coral health and functioning. Here, we review the roles these organisms play in the holobiont, including nutritional exchanges with the coral host and decalcification of the host skeleton. Microbial metabolism causes steep physico-chemical gradients in the skeleton, creating micro-niches that, along with dispersal limitation and priority effects, define the fine-scale microbial community assembly. Coral bleaching causes drastic changes in the skeletal microbiome, which can mitigate bleaching effects and promote coral survival during stress periods, but may also have detrimental effects. Finally, we discuss the idea that the skeleton may function as a microbial reservoir that can promote recolonization of the tissue microbiome following dysbiosis and help the coral holobiont return to homeostasis.

## The expanding field of coral skeleton microbial ecology

**Fig. 1:**
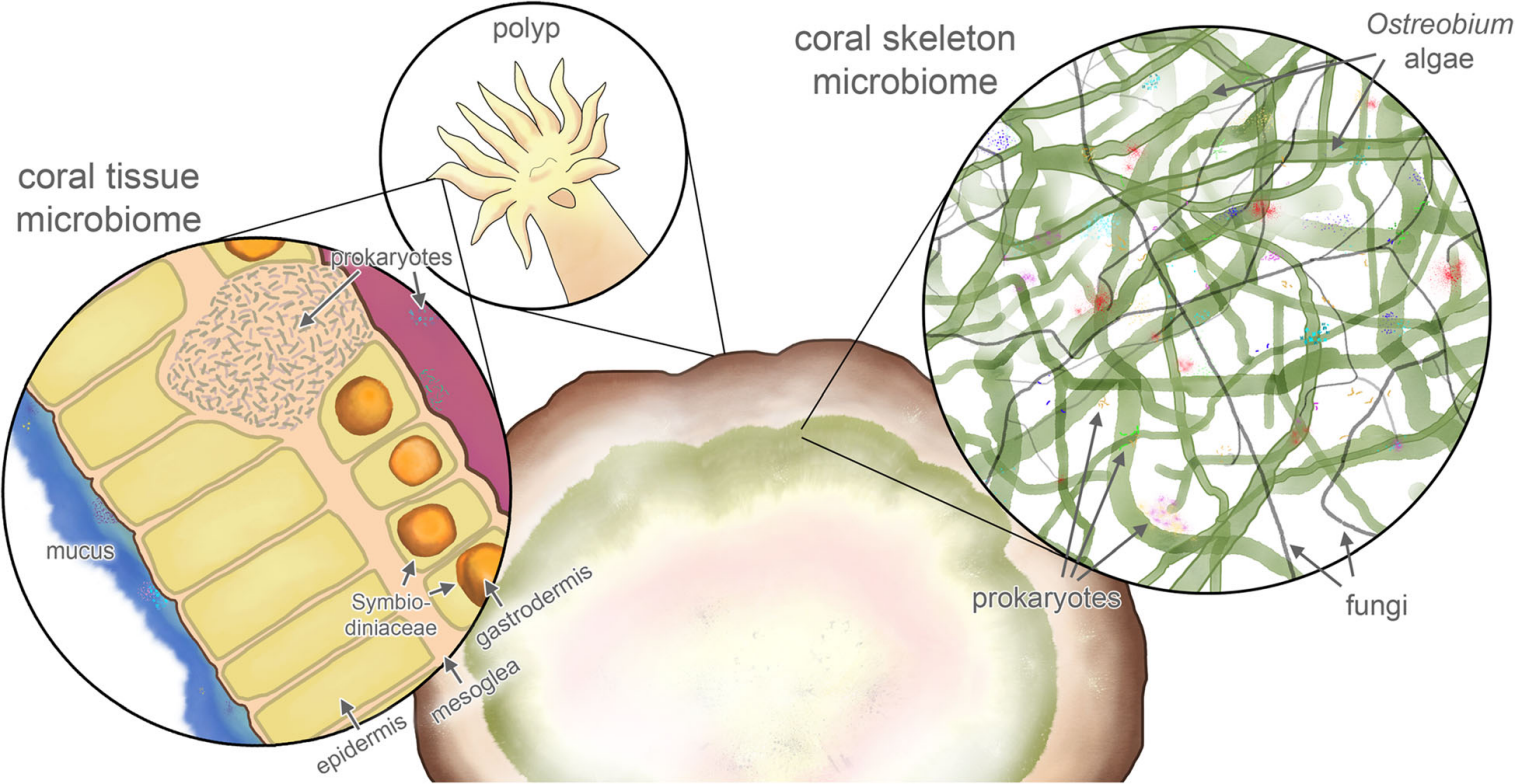
Cross-section of a coral skeleton, showing the microbiome associated with the tissue and mucus (left inset) and the skeleton (right inset). In the tissue, Symbiodiniaceae provide the coral with sugars through photosynthesis, and a rich prokaryotic microbiome is associated with mucus, epidermal and gastrodermal tissue layers, which often show bacterial aggregates. In the skeleton, a remarkably species-rich microbiome including the green alga *Ostreobium*, fungi and prokaryotes is found. The illustration depicts a relatively young massive coral species; older skeletons often have a more complex layered microbiome (see section "The skeleton as an environmental and biological record-keeper")

The coral holobiont comprises the coral polyps and a rich microbial community of prokaryotes, micro-eukaryotes and viruses (Fig. 1) [1, 2]. Anatomically, it consists of a thin film of mucus and tissue over a voluminous, porous calcium carbonate skeleton. The past decades have seen significant advances in our understanding of the diversity and roles of endosymbiotic algae (zooxanthellae in the family Symbiodiniaceae) and other microbes inhabiting the coral tissue and mucus [1, 3–5], while the microbiota residing in the skeleton have been side-lined. However, in the last few years, several studies have shed light on the ecology, biodiversity, physiology and metabolism of the skeletal microbiome, bringing its complexity into focus and raising hypotheses about its functions within the coral holobiont. Metabarcoding studies show a much higher biodiversity than anticipated, and a strong spatial structure of prokaryote distributions [6]. Ecological, physiological and more recently metagenomic studies are clarifying the functions of skeletal microbiota in the holobiont, including recycling of nutrients like nitrogen and sulphur [7, 8], providing alternative sources of energy [8, 9], decalcifying the skeletal matrix [10] and shaping the physico-chemical properties of the skeleton [11, 12]. This review aims to integrate ideas in the rapidly expanding field of coral skeleton microbial ecology and puts the fragmented information into an ecological context.

## Diversity and distribution of the skeletal microbiome

**Fig. 2:**
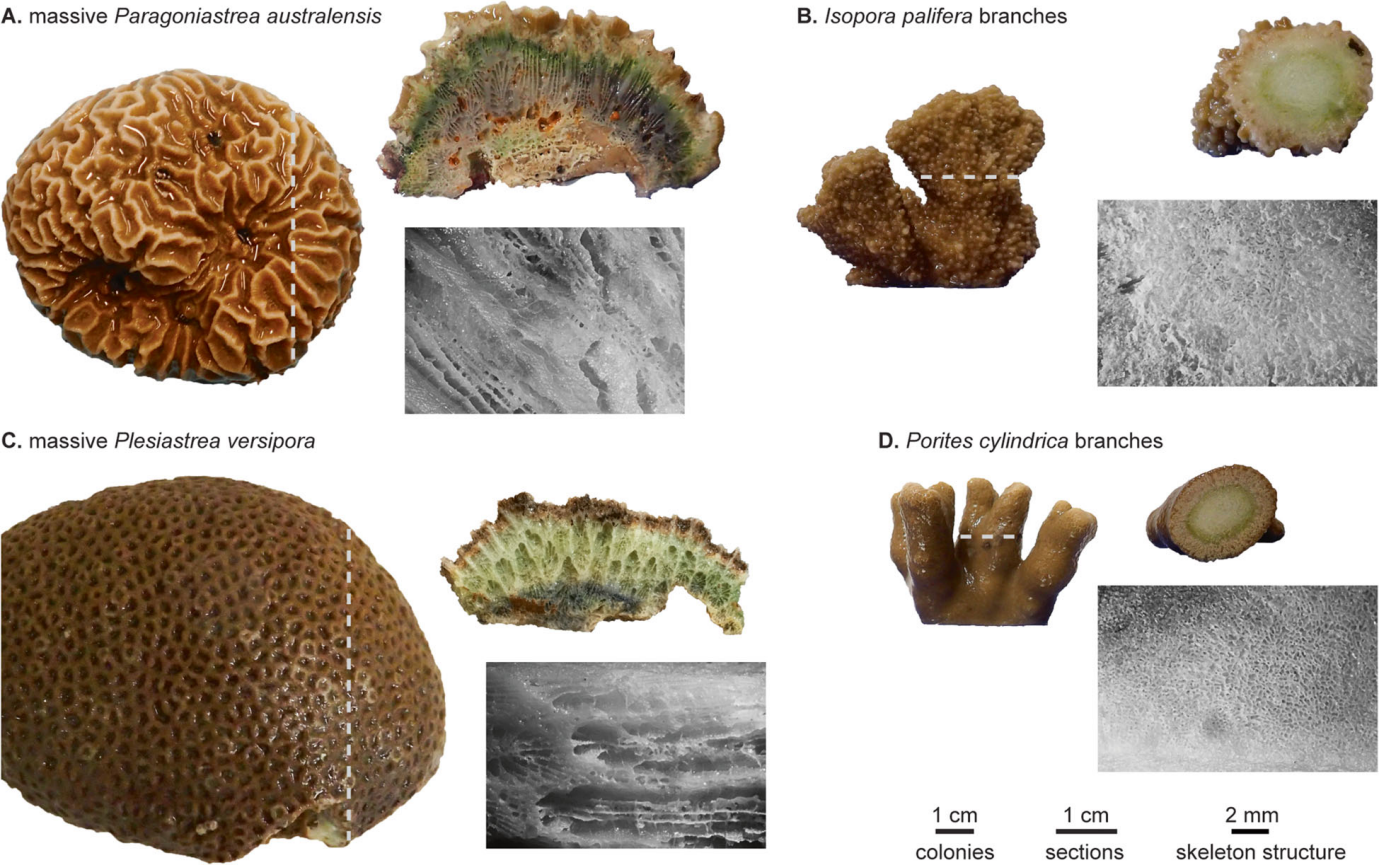
Colonies, skeletal cross sections, and microscopic images of the skeleton structure of four coral species. Dashed lines indicate the approximate cut orientation used to produce the sections. The cross-sections clearly show green colouration of the skeleton, indicating the presence of endolithic chlorophyll-containing phototrophs, and some skeletons show additional grey and orange regions indicative of other endolithic microbiota. The detail images of the skeletal structure illustrate that the two massive species illustrated (**a** and **c**) have large pores defined by the corallite structure, while *Isopora palifera* (**b**) and *Porites cylindrica* (**d**) have much smaller pore sizes and denser skeletons

Eukaryotic and prokaryotic microorganisms are very diverse and abundant across the CaCO_3_ skeleton (Figs. 1 and 2) [2, 6, 13–24] and are called endoliths due to their rock-dwelling nature. Among the eukaryotes are different groups of algae, sponges, fungi and protists, some of which actively bore into limestone, while others inhabit skeletal pores or cavities made by other microorganisms [2, 14, 25, 26]. Alpha- and Gammaproteobacteria are predominant bacterial classes [2, 27] and recent studies show that prokaryotes are even more diverse in the skeleton than in the coral tissue or mucus [28].

Eukaryotic green algae are abundant in the skeletons of live corals, exceeding the biomass of Symbiodiniaceae in the tissue by 16 times [29]. The green alga *Ostreobium* is the most common genus, present in the vast majority of stony coral samples [2, 29–31]. Its simple morphology and the laborious nature of isolating and culturing endolithic green algae has limited our knowledge about the biodiversity of these organisms, but culture-independent sequencing approaches have recently shown a massive biodiversity of green algal endoliths in coral skeletons, including a lineage of about 80 different *Ostreobium* species and several other entirely unknown family-level lineages [13, 24], suggesting that distantly related green algal lineages may have gone under the name “*Ostreobium*” in previous studies.

Fungi are also often reported in coral skeletons [2, 16, 17, 19, 25, 32]. They can feed on endolithic algae and coral polyps [15, 17] and are best known for their detrimental roles toward stony and soft corals [15–17, 33, 34]. Fungal hyphae growing toward coral tissue can be impeded or stopped by the host by encapsulating them in aragonite cones, preventing tissue perforation [18]. Fungi are also common in healthy corals and can coexist with the rest of the coral microbiome in a healthy equilibrium [15].

**Fig. 3:**
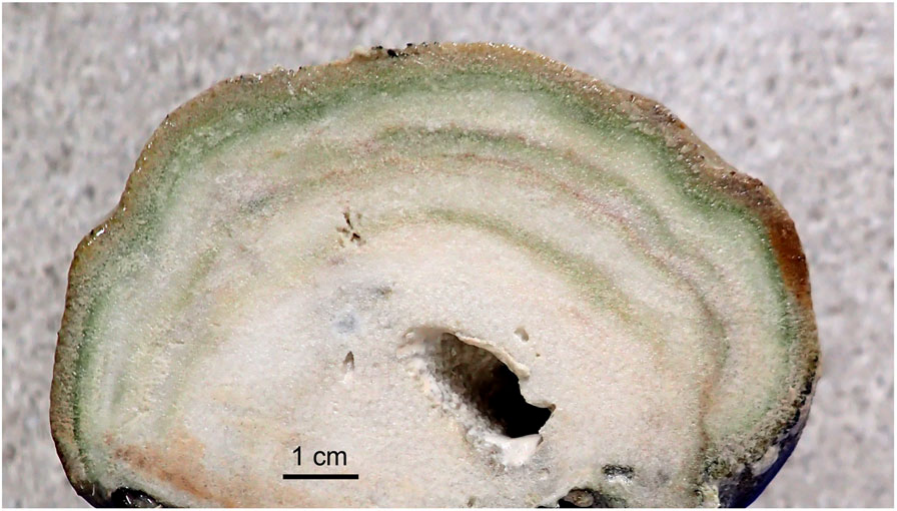
Cross-section through the skeleton of a small *Porites lutea* coral head showing bands of biogenic origin at multiple depths. For larger colonies, such bands may provide a decadal (or longer) record of coral biology and climate if the information can be accessed

The spatial distribution of the microbial biodiversity in the skeleton is an active area of research. Endolithic algae are ubiquitously present in tropical coral reefs [24], but have also been recorded in corals from high-latitude areas such as the Chilean fjords [35]. They occur in shallow and deeper waters (> 100 m) as well as in cave-dwelling corals [29, 31, 36, 37], and there is some evidence suggesting that the distribution of *Ostreobium* lineages is structured along a depth gradient [38]. At much smaller scales, there is strong patchiness in microbial distributions within individual colonies, where the prokaryotic community shows strong species turnover even at centimetre scales in the outer skeleton of individual colonies [6]. Endolithic algae, which actively tunnel their way through the skeletal matrix, show more homogeneous distributions in comparison to the prokaryotes [6]. It is likely that spatial differences also exist across the vertical axis of the coral colony, from its surface deeper down into the skeleton, as distinct green, grey and occasionally pink layers are visible to the naked eye (Figs. 1, 2 and 3) [16, 26, 39].

**Fig. 4:**
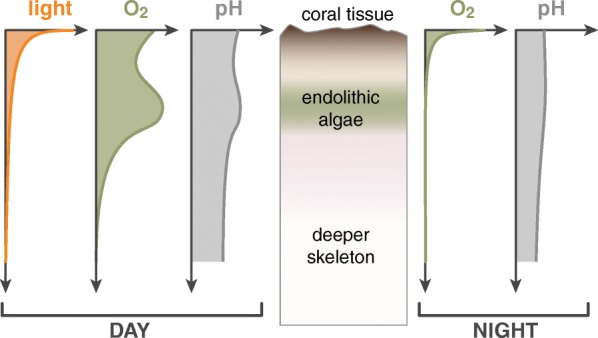
Micro-environmental gradients through a vertical cross-section of the skeleton of a massive coral species dominated by an *Ostreobium* algal band. During the day, sunlight gets depleted in the coral tissue with only small amounts of mostly far-red light reaching the skeleton. Oxygen levels are high near the tissue and in the endolithic algal zone due to photosynthesis, and this is also reflected in elevated pH in those zones. During the night, oxygen gets depleted from the skeleton by respiration and pH levels drop

Endolithic organisms are abundant in a variety of coral species, including massive species (Fig. 2a, c) and smaller branched forms (Fig. 2b, d). Like certain coral tissue-associated bacteria [40, 41], some endolithic microbes are consistently found in association with specific coral species [24]. For example, the endolithic community of massive *Porites* spp. was found to be distinct and more diverse when compared to the branching corals *Seriatopora hystrix* and *Pocillopora damicornis* [42], and a recent study found that the endolithic microbiome correlates with host phylogeny over a range of coral species [28]. Whether these correlations reflect species-specific interactions, phylosymbiosis, an effect of coral growth forms and skeletal microhabitats, or which combination of these, remains to be investigated in detail.

## Micro-niches in the skeleton

Scleractinian corals have diverse ecological micro-niches shaped by physico-chemical gradients across the various tissue and skeleton compartments (Fig. 4) [11, 12, 43]. These gradients are affected by the environment surrounding the coral holobiont [10, 12, 44], the skeletal microstructure [45, 46], and the physiology of holobiont members [11, 47]. Light is a crucial source of energy in this system, and the great majority of photosynthetically active radiation is absorbed by Symbiodiniaceae in the coral tissue [48] with only a small fraction penetrating into deeper layers (Fig. 4) [12, 43, 49, 50]. Inside the skeletons of shallow-water corals, mostly the far-red wavelengths (> 700 nm) remain [12]. These low-energy photons can be harvested by phototrophic endoliths through a variety of mechanisms including specialised pigments (chlorophylls d and f, bacteriochlorophylls) and uphill energy transfer [51–53]. Inside the skeleton, O_2_ production through photosynthesis is most pronounced in the green layer, creating a local O_2_ maximum (Fig. 4) [11]. Oxygen diffuses through the porous skeletal matrix into shallower and deeper parts of the skeleton, where it is consumed, and 1–2 cm below the maximum, the skeleton is completely anoxic [11]. The autotrophic metabolism of photosynthetic endoliths is also responsible for increasing pH in the medium, this process having a stronger influence on skeletal pH than the outside environment [43]. The dissolution process of CaCO3 which takes place both by day and at night, increases pH and the total alkalinity of the system [54].

Information on how skeleton architecture affects micro-niches is scarce, but the great variety of coral growth forms and skeletal features are likely to contribute to shaping the physicochemical characteristics of the colony. Light in the skeleton is scattered by the skeletal microstructure [55], with different coral species having been shown to have different scattering properties [46]. The association between skeletal structure and other physicochemical properties have not been investigated in detail, but one could hypothesise that porous and highly interconnected skeletons (e.g. Fig. 2a, c) may allow more diffusion of liquids and gases, leading to gentle environmental micro-gradients, while denser skeletons (e.g. Fig. 2b, d) may show opposite dynamics, with steep gradients driven by local biological processes (e.g. O_2_ peak corresponding to the endolithic algal layer, Fig. 4).

The micro-environments within the skeleton are subjected to a pronounced day/night cycle, being dominated by photosynthesis during the day and respiration during the night [43]. In daylight, O_2_ is produced and the environment becomes more alkaline due to CO_2_ removal, with pH values exceeding 8.5 (Fig. 4) [43]. The dominance of heterotrophic metabolisms during the night leads to quick consumption of the produced O_2_ (Fig. 4), shifting the environment to near-anoxia [11], and leads to a sharp drop of the pH by almost one pH unit [43]. After a few hours of darkness, the produced O_2_ is totally consumed in many skeletal zones. Anoxia promotes dinitrogen fixation by diazotrophic bacteria, although their activity in coral skeleton has been recorded even during the day [56].

## Community assembly processes

The recent progress in coral skeleton microbial ecology allows us to form a working hypothesis about community assembly and functioning in the skeleton. We propose that the skeleton sustains a highly diverse but functionally redundant microbial community that is shaped by micro-niche partitioning, priority effects and evolutionary associations.

Niche partitioning over different spatial and temporal scales is a commonly observed process that supports biodiversity by preventing competition among species and allowing their co-existence [57]. The coral skeleton certainly has multiple physico-chemical micro-niches, spatially across depth layers, and temporally during day/night cycles, seasons and life stages. These micro-niches sustain microorganisms from a range of functional groups, including aerobic and anaerobic bacteria, phototrophs, diazotrophs, decomposers, and microorganisms producing signalling metabolites and antimicrobial compounds [2, 15, 42, 56]. Limited dispersal is another factor that may contribute to the high biodiversity of coral skeletons. Prokaryotes living at equivalent depths inside the skeleton show a remarkably strong species turnover at centimetre scales [6]. In *Porites* for example, a coral skeleton fragment of ~ 0.23 cm^3^ contains about 25% of the prokaryotic diversity observed in the outer skeleton of the entire colony, indicating that microbial distribution is patchy even within ecologically homogeneous depth layers [6]. Spatial and temporal heterogeneity of environmental conditions promotes functional redundancy [58] and favours ecosystem stability [59, 60], which we expect to be important traits of the skeletal microbiome. We propose that environmental gradients and microbial interactions (e.g. competition, mutualism) in the limestone substrate result in patchy assemblages of microorganisms at very small spatial scales, characterised by high species diversity and functional redundancy in the skeletal microbiome of living corals.

Priority effects, the impacts that a species can have due to early colonisation of a habitat, likely play a strong role in shaping the species composition of the endolithic community, especially in young corals. A recent study reported *Ostreobium* in skeletons of coral recruits within the first week of larval settlement, and the algal colonisation increased with colony age, leading to a complex network of filaments of endolithic eukaryotes throughout the entire skeleton (Fig. 1) [44]. One can hypothesise that the first *Ostreobium* species to colonise and drill into the coral skeleton can shape their endolithic niche and likely remain predominant in the green endolithic layer through the life of the coral. A similar mechanism has been proposed to explain the assembly of the first gut microbiome in infants and its long-lasting effects on human health [61]. Besides priority effects, age-related changes in the endolithic community assembly are likely to occur. In fact, it has been shown that larger corals have a more diverse skeletal microbiome than smaller (and likely younger) colonies [62], and that prokaryotic species composition changes with the size of colonies [28]. These observations may reflect natural successional processes that occur over the course of coral development, and/or the ability of larger corals to sustain more diverse micro-niches. External factors may also play a role, including climate anomalies, physical disturbances (e.g. parrotfish grazing) and other factors that may transform community assembly patterns in the skeleton [44, 63]. The most extreme example is seen during coral bleaching and death, when photobionts come to dominate the endolithic community [64, 65].

Phototrophs are likely to be keystone organisms in the endolithic community, given their disproportionally important role in community assembly. The anaerobic photoautotrophic *Prosthecochloris*, a dinitrogen-fixing green sulphur bacteria, is predominant in *Isopora* corals [14], while they are only present in low relative abundances in other corals dominated by *Ostreobium* [2, 42]. In contrast to an *Ostreobium*-dominated environment, the anoxygenic photosynthetic green sulphur bacteria only thrive in an oxygen-poor skeletal environment, and have strong effects on nitrogen and sulphur cycling via their metabolism, which has downstream effects on the assembly of the skeletal microbiome [7]. An active field of research is to determine whether recently discovered endolithic lineages (e.g. different *Ostreobium* species and bacterial lineages) have distinct eco-physiological traits—and hence may trigger distinct community assembly patterns—and which ones are functionally redundant.

## The skeleton as an environmental and biological record-keeper

Because the rate and density of CaCO_3_ deposition by corals varies seasonally, the skeleton has easily recognisable growth rings similar to those produced by trees on land [66]. Like in trees, these rings can be used to estimate colony age and growth rate, and thus serve as a record for historical climate as well as coral bleaching [67, 68]. Coral skeletons have been used for a long time as a record-keeper of environmental information. Long-lived massive corals are a reliable proxy for historical pH fluctuations (Reynaud et al. 2004), which suggest that natural ~ 50 years pH oscillation could mitigate the impact of ocean acidification on coral reef ecosystems [69]. Stable isotopes of skeletal materials have been used to reconstruct past variations of sea surface temperature [70] and to study the influence of anomalous climatic oscillation (e.g. El Niño/Southern Oscillation) on coral biology [71]. Recent work has shown that some coral species are more suitable than others to serve as a climate proxy, and that data are influenced by the biology of the chosen specimen [72]. Skeletal intra-crystalline δ^15^N has been used to trace excess nutrient loading in reef ecosystem [73], which, coupled with low pH, can enhance corals’ sensitivity to bioerosion [74]. δ^15^N measurements can be a useful tool to distinguish among different nitrogen sources and shed light on the impact of anthropogenic nitrogen fluxes into reef ecosystems. Interestingly, besides this historical environmental record, older corals can also show multiple coloured bands of biological origin in their skeleton, with deeper greenish or greyish bands in addition to the green band just underneath the coral tissue (Fig. 3). At least in some corals, these coloured bands correspond to annual growth patterns [75]. The deeper bands are likely remnants of past blooms of endolithic algae, often consumed by fungi giving them a greyish colour [16, 75], while it is not always clear what deeper green bands represent. One could hypothesise that they are dead algae not yet consumed by fungi, but it is also possible that some of these bands harbour live phototrophic microorganisms specialised in different light environments. Green photosynthetic bacteria, for example, are known to thrive in extreme shade due to efficient light harvesting by their chlorosomes [76]. In any case, the skeleton is a complex structure, where niche differentiation along microenvironmental gradients appears to be superimposed upon a historical record of biological processes that spans decades, potentially even centuries in old colonies. Developing the appropriate methods to probe this record of biological processes and environmental conditions over time (e.g. by ancient DNA analysis, isotopic measurements, pigment analysis, and culturing microbiota from different bands) has the potential to shed light on the increasing impact of bleaching and human activities on coral holobionts.

## Implications for the coral

### Nutritional exchanges

Corals rely on nutritional interactions with their microbiome to succeed in oligotrophic environments. The endolithic community may participate in the holobiont metabolism by providing and recycling substrates and nutrients. Organic compounds produced and excreted by photosynthetic organisms (and potentially other autotrophs) in the skeleton can fuel other members of the holobiont including fungi and other heterotrophic endoliths [15]. Importantly, carbohydrates produced in a green *Ostreobium* band were shown to be translocated to and incorporated into the coral tissue [77]. While the nature of the translocation is unclear, it suggests an established metabolic interaction between endolithic algae and corals [78].

The skeleton has also been proposed as an important site of inorganic nutrient regeneration. It is well known that, in comparison to the surrounding environment, the skeletal pore water is enriched in labile nitrogen and phosphorus species [79, 80], and that active dinitrogen-fixing bacteria are present in the skeleton [56]. A recent metagenomic study revealed a wide range of genes involved in nitrogen and sulphur biochemical pathways in the endolithic microbiome, and it is likely that such metabolic functions can be carried out by many skeletal bacteria [7, 8, 14]. Oxygen availability is also important to the coral and its microbiota [81], and the dynamics of this element in the skeleton is largely determined by algal and cyanobacterial photosynthesis and microbial respiration [11]. The degree to which O_2_ and other nutrients are recycled within the skeleton and exchanged with the coral remains to be determined, but studies showing that carbon and nitrogen get transferred toward to the coral tissue [8, 9, 77] suggest that the skeleton is a source of nutrients for the coral.

### Skeletal decalcification

The structure of corals and the entire reef system depends on the balance of calcification and decalcification, both of which are strongly biologically mediated [45, 82]. Many endolithic organisms including cyanobacteria, algae and fungi contribute to bioerosion of the coral skeleton [30, 82, 83], but the green alga *Ostreobium* is by far the most important agent of skeletal deterioration, responsible for 60–90% of microbial carbonate removal in coral skeleton [30, 65]. Most quantitative information on bioerosion comes from experiments on dead coral skeleton [30, 54], where microbial bioerosion rates are significant: up to 1.1 kg CaCO_3_ dissolution per m^2^ of exposed substrate area per year [26], corresponding to about 20% of the annual CaCO_3_ production in coral reefs [54]. Much less knowledge is available for live corals, although it is known that less skeletal burrowing takes place in living corals than in dead carbonate skeletons. Nonetheless, microborers are present throughout the skeleton in living corals from very early developmental stages [44], and in the more densely populated areas of mature skeletons, more than 25% of the skeletal volume is occupied by microborers [26], implying substantial decalcification of live coral skeletons. Microbial bioerosion is known to increase at higher temperatures and lower pH, and it has been estimated that by the year 2100, coral endoliths will dissolve ca. 70% of the yearly reef CaCO_3_ production [54, 84], suggesting that this process will contribute to accelerated reef deterioration (and possibly coral fragility) in future ocean conditions [10, 84].

### Diversity-mediated coral health and resilience

During disease, the coral microbiome composition shifts from homeostasis to a state of dysbiosis [3, 85, 86]. This shift is often triggered by environmental stressors like high temperature, and from a microbial perspective is characterised by a reduced population of beneficial species and a higher abundance of potentially harmful ones, some of which reside in the skeleton. For example, endolithic green algae bloom during coral bleaching and coral white syndrome in response to deeper light penetration into the skeleton [54, 87]. Since endolithic algae have been reported penetrating and can apparently cause lesions of the coral tissue [87, 88], it seems possible that the increase in algal biomass during coral bleaching can affect the health status of the coral animal and its susceptibility to pathogens. Data also suggest the coral skeleton as a potential reservoir of the cyanobacterium *Phormidium corallyictum* causing the deadly black band disease [89].

Besides potential pathogens, the coral skeleton may also harbour beneficial microorganisms playing a role in coral resilience and recovery from disturbances—a proposition known as the skeleton reservoir hypothesis [6, 42]. Many microorganisms that are regarded as functionally important in coral tissues (e.g. *Endozoicomonas* spp.) also occur in coral skeletons [2, 23, 62, 90]. The notion that biodiversity begets stability is a central principle in ecology [91, 92], suggesting that the highly diverse skeletal ecosystem should be more resilient to stressors than other parts of the coral and potentially act as a safe-house for coral microbiota. In fact, it has been demonstrated that the microbial community present in coral skeletons is more resilient to high *p*CO_2_ than that in coral tissues [42]. Symbiodiniaceae play an important role in maintaining pH homeostasis in the coral tissue [93], and it is possible that the abundant endolithic algae contribute to the buffering capacity of the skeletal environment [54]. Analogous to the human appendix, which serves as a refuge for gut microorganisms that re-populate the colon after illnesses [94], the skeleton may also play a role in assisting the recovery of the coral microbiome during and after diseases, although this hypothesis still needs validation. Computational modelling is particularly promising to determine which (and when) members of the microbiome have key roles in health and resilience (e.g. [95]).

The manipulation of the coral-associated microbiome has been proposed as a promising approach to improve coral tolerance to stress [96–98]. The inoculation of corals with probiotics isolated from coral tissue and surrounding water has been shown to reduce the susceptibility of corals to temperature-induced bleaching [98]. Additionally, genetic engineering targeting the thermotolerance of key symbionts can also enhance the resilience of corals to climate change [99]. Probiotics and genetic engineering may provide a rapid and urgently needed response to coral decline, but it must be noted that the field is in its infancy and substantially more research is needed to understand its efficacy and risks [100]. The potential of using beneficial endolithic microorganisms as probiotics, or the long-term effects of manipulating members of the coral microbiome on the endolithic community, is yet to be studied in detail.

### Bleaching of the coral, greening of the skeleton

Heat stress transforms the physico-chemical properties and biology of the coral holobiont and when a coral bleaches, the whole colony including its skeletal microbiota is affected [101]. During bleaching, the symbiosis between the coral and Symbiodiniaceae breaks down, and the latter are degraded or leave their host [102]. Without the Symbiodiniaceae absorbing light and consuming CO_2_ in the coral tissue, more of the solar irradiance and CO_2_ is likely to reach the skeleton, which may contribute to fuelling photosynthesis by the endolithic algae that bloom during bleaching events [9, 43, 64, 87].

It has been hypothesised that the endolithic community may protect the corals and help them overcome bleaching periods [9]. During bleaching, the increased light scattering from the skeleton affects any remaining Symbiodiniaceae and can accelerate bleaching—a mechanism known as the optical feedback loop [103, 104]. By absorbing more light, blooming endolithic algae colonising the outer portions of the skeleton may reduce the light scattering from the skeleton, alleviating photic stress for the coral and the remaining Symbiodiniaceae [55, 105]. Furthermore, photosynthates excreted by *Ostreobium* may be transferred to the coral animal [8, 77]. Such translocation appears to be enhanced during bleaching events, which may alleviate energy limitation and promote recovery of the coral animal during a temporary loss of Symbiodiniaceae [9]. While the translocation mechanism is unknown, it may be aided by *Ostreobium* filaments growing toward the coral tissue.

Bleaching-induced stimulation of endolithic photosynthesis is likely to affect the physico-chemical conditions in the skeleton, with downstream effects on microbiota and the coral animal, but little is known about these processes. We hypothesise that the O_2_ gradient (Fig. 4) will intensify due to increased photosynthesis, also leading to stronger diurnal pH fluctuations. It also seems likely that carbohydrates produced by endolithic photosynthesis will become available to other members of the microbiome. We hypothesise that this will lead to changes in the community composition and function of other microbes and may also stimulate the development of diverse pathogens. Metagenomic work on coral tissue has indeed shown that the microbiome of bleached corals is enriched in carbohydrate processing and there is an increase in virulence-associated genes [101, 106], but the causes for these changes are not fully understood and the link between processes happening in the skeleton and the coral tissue is yet to be studied in detail.

There is no doubt that bleaching has a severe influence on the coral holobiont and its microbiome constituents. While such stress-induced changes in the composition and functioning of microbiomes are starting to be understood for the coral tissue, many questions remain about what happens in the skeleton. The endolithic algal blooms can play a potentially important role in photoprotection and probably in transfer of photosynthates to the coral animal during and after bleaching events. However, the actual changes in physico-chemical microenvironments and skeletal microbiome structure have not been described, and detailed information about interactions between endolithic microbes and the coral host is not currently available. Because of the likely importance of the skeletal microbiome, we consider this a very promising avenue for future research.

## Conclusions

This review highlights that the coral skeleton is much more than the mere structural support of corals. It is a key compartment of the coral holobiont that harbours a diverse and highly structured microbial community that can affect the coral in various ways. While our knowledge about this aspect of the holobiont has grown substantially in the past few years, many open questions remain. Characterising the roles performed by the various endolithic microbial species and their relevance in the holobiont remains a difficult task given the challenges involved in studying rock-dwelling organisms, the vast number of microbial species involved and the sparse information available. How do skeletal micro-niches vary across coral species and with skeletal traits (e.g. coral morphology and density)? What changes in microbial community structure and function occur during the *Ostreobium* bloom that follows coral bleaching, and what is the net effect of beneficial and detrimental effects on the coral? What are the eco-physiological differences between cryptic endolithic algal lineages, and those that bloom during coral bleaching? What is the relative contribution of the skeletal microbiome to nutrient cycling and coral nutrition during coral health and disease, and which skeletal microbes play key roles for the coral animal? Is there co-evolution between coral and endoliths, or do ecological processes suffice to explain the correlation between endolithic community composition and coral phylogeny? We hope that by providing an overview of the current knowledge on the coral skeleton, and identifying knowledge gaps, this paper will stimulate further research into this hidden, yet important microbial reef habitat.

## Data Availability

Not applicable

## Abbreviation

DNA: Deoxyribonucleic acid
